# Missed fractures in severely injured patients

**DOI:** 10.1186/cc14392

**Published:** 2015-03-16

**Authors:** H Park

**Affiliations:** 1 Dankook University Hospital, Cheonan City, South Korea

## Introduction

The purpose of this study is to analyze anatomic distributions, diagnostic methods, and prognosis of missed fractures in patients with severe injury.

## Methods

A review of single-institutional medical records between January 2001 and May 2012 identified 58 patients with 62 delayed diagnoses of fractures among 4,643 severely injured patients older than 20 years with Injury Severity Scores higher than 16. We evaluated combined injuries, location of fractures, diagnostic methods, and reasons for missed diagnosis at initial examination.

## Results

Among 62 missed fractures, there were eight cases of spine fracture, 10 cases of peri-shoulder joint fracture, eight cases of upper extremity fracture, 10 cases of pelvis of acetabulum fracture, and 26 cases of lower extremity fracture. Head injury was the most common concomitant injury (23 cases). Initially missed fractures were most commonly discovered by official reading by radiologists. The most common reasons for misdiagnosis were the use of improper radiologic study and missed-reading of proper radiologic studies.

## Conclusion

In order to prevent misdiagnosis of fractures in patients with severe injury, meticulous physical examination with suspicion of fractures should come first. In addition, obtaining proper radiologic study and thorough evaluation of radiologic images are important to decreasing the rates of missed fracture diagnoses. In addition, thorough surveillance for ipsilateral fractures is important in extremities with identified fractures.
